# Biofilm and swarming emergent behaviours controlled through the aid of biophysical understanding and tools

**DOI:** 10.1042/BST20200972

**Published:** 2020-12-10

**Authors:** Iago Grobas, Dario G. Bazzoli, Munehiro Asally

**Affiliations:** 1 School of Life Sciences, University of Warwick, Coventry CV4 7AL, U.K.; 2 Warwick Medical School, University of Warwick, Coventry CV4 7AL, U.K.; 3 School of Chemical Engineering, University of Birmingham, Birmingham B15 2TT, U.K.; 4 Warwick Integrative Synthetic Biology Centre, University of Warwick, Coventry CV4 7AL, U.K.; 5 Bio-Electrical Engineering Innovation Hub, University of Warwick, Coventry CV4 7AL, U.K.

**Keywords:** active matter, biofilms, living materials, pattern engineering, physics of microbes, swarming

## Abstract

Bacteria can organise themselves into communities in the forms of biofilms and swarms. Through chemical and physical interactions between cells, these communities exhibit emergent properties that individual cells alone do not have. While bacterial communities have been mainly studied in the context of biochemistry and molecular biology, recent years have seen rapid advancements in the biophysical understanding of emergent phenomena through physical interactions in biofilms and swarms. Moreover, new technologies to control bacterial emergent behaviours by physical means are emerging in synthetic biology. Such technologies are particularly promising for developing engineered living materials (ELM) and devices and controlling contamination and biofouling. In this minireview, we overview recent studies unveiling physical and mechanical cues that trigger and affect swarming and biofilm development. In particular, we focus on cell shape, motion and density as the key parameters for mechanical cell–cell interactions within a community. We then showcase recent studies that use physical stimuli for patterning bacterial communities, altering collective behaviours and preventing biofilm formation. Finally, we discuss the future potential extension of biophysical and bioengineering research on microbial communities through computational modelling and deeper investigation of mechano-electrophysiological coupling.

## Introduction

Emergent behaviours are those that arise through local interactions between constituents but cannot be explained reductively [1]. Bacterial biofilms and swarms are examples of such; these communities show properties that individual cells lack on their own. More specifically, bacterial communities can exhibit collective tolerance to environmental stresses [2–4], up-regulation of the secondary-metabolite synthetic pathways [5,6], division of labours [7,8] and collective information processing [9,10]. These emergent properties are not only of fundamental importance to the biological understanding of bacterial communities but also opportunities for innovations and applications to synthetic biology. For example, biofilms’ high resilience, long-term activity and extracellular poly-substances (EPS) can be exploited for high-value chemical production [11] and bioremediation [12]. In material science, biofilms provide a platform for developing a new class of materials, now known as engineered living materials (ELM) [13–16]. In engineering, swarming bacteria can be used for transporting micro-objects, such as living microbes, nanorods and microbeads [17–19]. Bacterial swarms can also serve as a model system for decentralised collective behaviours and swarm intelligence [20,21]. On the other hand, preventing the formation of bacterial communities is a key challenge in industrial and biomedical processes since biofilms are involved in biofouling, biocorrosion and contamination of medical devices while swarms can be associated with pathogenesis and infections [22,23]. Therefore, understanding the mechanisms by which emergent properties of bacterial communities arise is an important research topic in a broad range of research fields, including microbiology, biophysics, material sciences, bioengineering and synthetic biology.

In the past, studies into bacterial communities have mainly focused on the characterisation of biochemical and molecular mechanisms [24]. However, the last decade has seen a rapid advancement in the biophysical characterisation of microbial emergent dynamics [25–27]. It is now evident that not only the biochemical and genetic pathways, but also the physical interactions — e.g. cell cohesion, mechanical buckling and electrical signalling —play important roles in regulating the complex dynamic behaviours of biofilms and swarms [28,29]. Importantly, biophysical studies of bacterial communities can lead to novel tools, techniques and approaches to engineering and controlling beneficial biofilms and swarms in space and time and preventing harmful ones.

**Figure 1.**
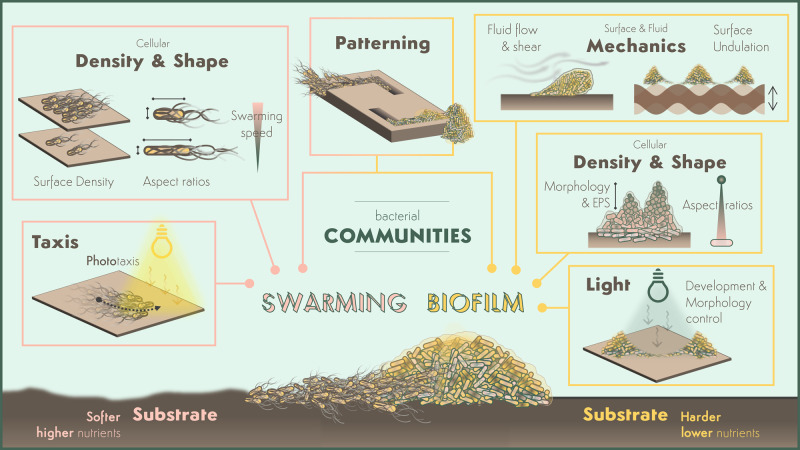
Different Physical properties can regulate microbial swarming (left) and biofilm (right) collective behaviour and development. Soft substrates that are rich in nutrients promote swarming bacteria formation (left) whereas hard substrates and, in general, nutrient depletion promotes biofilm formation (right). Swarming motility is enhanced by the cell density and short aspect ratios. In biofilms, cells of large aspect ratios are found at the bottom and rounded cells in upper layers and the differences in cell density provide the biofilms’ typical wrinkle-like structure. Light can drive the expanding direction swarms in phototactic bacteria and tune their cell speed leading to patterns and inhomogeneities in cell density. In biofilms, light can be used to control single cell attachment in synthetic engineered bacteria or to trigger biofilm dispersion through cell hyperpolarization. Biofilm inhibition is also possible by surface undulation at certain amplitude and frequency and its shape and degree of attachment can be altered by external shear flows. Both biofilms and swarms can be organised into arbitrary patterns through 3D printing and MeniFluidics. See also accompanied table.

In this minireview, we overview recent biophysical and synthetic-biology studies on bacterial communities (Table 1 and Figure 1). We first briefly overview the physical environmental conditions that affect bacterial collective behaviours. We then showcase recent findings of key biophysical cellular properties for bacterial collectives and emerging techniques for controlling biofilms and swarms. We note that, for the sake of brevity, this minireview does not cover the biochemical and genetic characterisations and applications of bacterial swarms and biofilms. For those topics, we encourage the readers to read relevant reviews (e.g. [13–16,30,31]). We also refer readers to the reviews on social and evolutional interactions in bacterial collectives [32,33], as they are highly relevant but beyond our scope here.

**Table 1.** Summary table

	Swarm	Biofilm
Substrate	Semi-solid (0.3–1% agar)	Liquid, solid (1.5% agar)
Cell aspect ratio(AR)	AR 5 for optimal swarming motility [50]	Rod-shaped cells at the base of the biofilm and rounded cells in the upper layers [55]
Cell density	Minimal cell density required for swarming. Increasing cell densities enhance bacteria motility [18,50]	Inhomogeneous cell density patterns of living and dead cells shape biofilm's wrinkle architecture [58] and EPS production is thickness dependent [60]
Light	Light drives bulk swarm motility in phototactic bacteria and blue light can slow down cells and lead to local cell accumulation [65,66]	Blue light can be used to pattern biofilms in genetically engineered cells or to induce biofilm dispersal through cell hyperpolarization [74–77,79]
Mechanical patterning	Geometric confinement induces vortex patterns useful to identify swarming motility [70,71]	3D printing and Menifluidics can direct biofilm growth with submillimetre precision [72,81–83]
Mechanical surface waves		Tuning frequency and amplitude of surface waves can control biofilm formation [85–90,92]
Shear flows		High shear flows lead to more compact biofilm formation and changes in its mechanical properties [48,92,93,103]

## Both swarms and biofilms form on surfaces, but on surfaces with different mechanical properties

Swarming is a collective and most rapid mode of surface motility where motile cells are tightly packed and form dynamic motile rafts [34]. The centre of a swarming colony is typically formed by immotile or poorly motile cells [35,36], which is sometimes referred to as biofilm state [18,36]. However, whether these immotile cells express biofilm-related genes is unclear. Swarming is typically observed on soft (elastic modulus 20–100 kPa [37]) moist surfaces. In laboratory experiments, swarming dynamics can be induced by inoculating cells on a soft agar plate, typically between 0.3% and 1.0% (w/v), depending on the species [34]. When agar concentration is lower (<0.3%), cells swim, instead of swarm, through the porous agar. The water surface tension is a key determining factor for bacterial swarming behaviour as it determines the force required for cells to deform the air–water interface and spread on a surface [38]. Swarming cells are able to reduce the surface tension by secreting biosurfactants, such as surfactin in *Bacillus subtilis* [34] and serrawettin in *Serratia marcescens* [39].

Biofilms, sessile microbial communities embedded in EPS, also emerge on surfaces, but the conditions favourable for biofilm formation are distinct from those for swarming. Biofilms are prominent on solid–liquid, solid–air and liquid–air phase boundaries, and are recalcitrant to almost all types of biological and biochemical stresses. In the case of biofilm formation at solid-liquid interfaces, adhesion is the initial step, which is a complex process involving various physico-chemical factors and is mediated by van-der-Waals and electrostatic interactions between cells and surfaces [40,41]. Positively charged surfaces, which can result from the coating by positively charged compounds such as poly-l-lysis and APTES, promote the cell adhesion due to the fact that cellular surfaces are negatively charged [42]. Cell attachment can also be enhanced by decreasing hydrodynamic shear forces, hence local flow fields influence the attachment [43,44]. As cells proliferate at phase interfaces, cells secrete EPS, a mix of proteins, polysaccharides and DNA [45], which can act as a diffusion barrier [46], help water retention [47], adsorb ions [46] and provide structural integrity and mechanical stiffness (Young's modulus, 80–172 kPa [48]).

## Cell shape and density impact bacterial collective behaviour

Swarming and biofilm dynamics depend on their individual constitutive units, the cells. Their shape, motion, growth and density are important for their mechanical interactions and hence the emergent behaviours of swarms and biofilms.

### Swarm

Swirling patterns in swarms, lasting for several seconds, are inherent to systems composed of rod-shaped active particles [18,49]. Therefore, cell shape, more specifically the aspect ratio (ratio between the width and the length of the cells), is one of the key parameters that fundamentally define swarming dynamics [50]. In *B. subtilis* swarms, low aspect ratios (from 1 to 9) enable the normal swarming behaviour, characterised by a unimodal distribution of surface densities and a Gaussian distribution of the velocities (kurtosis close to 3). When the aspect ratio is large (>10), the swarm splits in two subpopulations of low- and high-density regions. The velocity distribution gives very large kurtosis (indicating heavy-tailed distributions) unlike in the small aspect ratio phase. The aspect ratio also affects the magnitude of the velocity, peaking at 50 μm/s (aspect ratio of 5) [50], which decreases by nearly 5-fold when the aspect ratio is changed to 3.8 or 8.

Changes in aspect ratio are also linked to the surface density of cells in the swarm and certain combinations of cell shapes and cell densities can enhance or even prohibit swarming motility. At surface coverages lower than ∼0.15 (15%), cells are practically immotile, suggesting a minimal surface density below which cells are unable to move. This bottom threshold is slightly reduced by increasing aspect ratios, suggesting that high aspect ratio promotes swarming at low surface densities. Increasing surface densities up to a threshold of ∼0.8 enhances swarming motility but, for higher values, cells stop moving efficiently and form a jam phase [18,50]. This threshold again depends on the aspect ratio since larger aspect ratios permit swarming at surface coverage slightly greater than 0.8.

The swirling motion characteristic of the rod-like cell shape makes the swarm super diffusive. Super diffusivity in a 2D system happens when the mean square displacement (MSD) follows a power law with an exponent greater than 1. In *B. subtilis* and *S. marcescens* swarms, this exponent is 1.6 [49]. The higher the surface density the less super diffusive the swarm is, a dependence that appears to be more sensitive for high aspect ratios: the diffusion exponent goes from ∼1.8 to 1.3 for aspect ratio of 19 or 13 and from ∼1.7 to 1.6 for aspect ratio 7 or 5.5. Since bacterial collectives use this super diffusivity to actively mix nutrients and oxygen [51], an increase in surface density might make cells more vulnerable to antibiotics. From a biological perspective, cell density and aspect ratio, constrain, or even control cells’ ability to move and spread collectively and efficiently.

Some of the collective dynamics allow swarming bacteria to better cope with environmental stress. In a recent study, we showed that swarming *B. subtilis* colony can undergo biofilm formation through dynamic localised phase transition, which allows swarming bacteria to overcome several forms of environmental stress such as antibiotics, UV light and spatial confinement [52]. This phase transition appeared to be compatible with a physical theory of collective motion, known as motility-induced phase separation (MIPS), where self-propelled particles (e.g. motile cells) could phase separate, i.e. segregate in two different states with different physical properties, into liquid-like and solid-like phases depending on the particle speeds and surface coverage [53]. Detailed further investigation into the phase transition is an important step forward towards understanding the biophysical mechanisms of collective cell differentiation into biofilms from swarms. Interestingly, in presence of antibiotic, swarms can cluster into spatially segregated phases of fast and slow rafts [54]. The clusters of faster cells, less affected by the antibiotic, are dominant at the swarming edge while the slower and more affected cells are left behind. This separation in motility phases enables swarming cells to colonise the regions with higher antibiotic level than they could on solid agar [54].

### Biofilm

In biofilms, cell-aspect ratio guides self-organisation into layered-structures, thereby providing particular genotypes with preferential access to favourable positions *in Escherichia coli* biofilms [55]. Rod-shaped cells colonise mainly the base of the community and its expanding edges, whereas rounded cells dominate the upper layers. A computational simulation of biofilm formation with cells of varying birth aspect ratio ranging from 1.1 to 3 agrees with the experimental observation [55]. The authors suggested that layering different-shaped cells could give rise to 3% better fitness when exposed to extreme conditions [55]. While the reason for this is not entirely clear, one possible explanation may be linked to oxygen availability.

Wrinkles are a characteristic morphological feature of air-interfacing biofilms, either at liquid–air (pellicles) or solid–air boundaries (colony biofilms). The biofilm wrinkles can act as fluidic channels [56] and increase the surface hydrophobicity [57]. Biofilm wrinkle formation is fundamentally a mechanical process [58] and, indeed, the characteristic wavelength of wrinkles correlates with the biofilms’ mechanical stiffness and thickness [59]. Both depend on the production of EPS, itself a function of the nutrient availability and biofilm thickness. In nutritious media, matrix production genes are up-regulated only beyond a height of 500 μm. However, in depleted media, matrix production starts at 250 μm [60]. Bacteria organise themselves within this varying thickness grouping in different layers which have implications in their aerobic state. The top layers are exposed to oxygen and therefore composed of fast-growing cells, whereas the layers located near the substrate are developed in an anaerobic state [61]. The slow growth rate of cells inside biofilms leads for instance to an enhanced tolerance to some antibiotics [62].

## Emerging techniques using physical stimuli for controlling swarms and biofilms

### Swarm

A physical entity that can be used as a tool for controlling cell-density heterogeneity in space and time is light. It can act on cells through phototaxis, negative or positive [63,64], or by slowing down motility through light-induced membrane depolarisation [65]. Infrared and blue-green light has been used to direct and repel swarming *Rhodospirillum centenum*, respectively [66]. Exposing a local region of swarming *S. marcescens* to light for <1 min can drop the average motility speed from ∼35 to 0 μm/s, depending on the swarming state and the light intensity [65]. The resultant local immotile population impedes the penetration of unexposed bacteria into the region, thereby reducing total damage to the whole colony [65]. Post-exposure, motile cells coming from the non-illuminated area disperse and push immotile bacteria away until the normal swarming motility is restored [65]. The time scale of photoreceptor activation and deactivation is important in the wavelength dependence [67,68]. Genetically engineering photo-sensor proteins is also a promising approach towards establishing light-control systems. Engineering proteorhodopsin into *E. coli*, dynamic cell density of swimming cells could be patterned by light [69]. A bacterial light-oxygen-voltage protein, EL222, has also been used for patterning swimming cells using blue light [63]. Extending these genetic tools to swarms is an exciting future research topic that may allow implementing more complex patterns for dynamic ELM.

Spatial confinement is another physical approach for inducing dynamic cell-density patterns, such as vortexes. A theoretical model of active fluidics has suggested that the confinement geometry could result in dynamic patterns, including vortex lattices [70,71]. While a detailed analysis is yet to be done, a similar pattern has been observed in swarming *B. subtilis* in a meniscus open channel, named MeniFluidics [72]. Such theoretical models and experimental techniques for engineering dynamic swarming patterns could lead to the developments of active-liquid metamaterials [73] and to identify swarming bacteria in physiological conditions [72]. The latter represents a challenge since swarming is mainly observed on agar surfaces.

### Biofilm

Light can be used also for controlling biofilms. With the aim of developing ELM, the technology called ‘biofilm lithography’ uses optogenetic tools to induce the planktonic-to-biofilm phenotypic switch, enabling spatio-temporal control of biofilm formation [74–77]. For example, the expression of antigen43 (Ag43), a cell–cell adhesion and substrate-attachment promoting factor, has been regulated by mild blue light using pDawn to optically control adhesion [74]. Recently, blue light has also been used for encoding memory in a dynamically oscillating *B. subtilis* biofilms [78]. Exposure to blue light causes a permanent shift in the phase of the membrane potential oscillation in the biofilm, which enables creating a permanent out-of-phase regions with arbitrary patterns. At higher doses (>120 μW/cm^2^), blue-light-induced hyperpolarization can disperse biofilms in *B. subtilis* and *P. aeruginosa* [79]*.* Kahl et al. [80] also showed that prolonged low-intensity blue light inhibits biofilm matrix production in *P. aeruginosa*, likely due to a reduction in c-di-GMP levels.

Another approach to create patterns within biofilms is 3D printing that uses a nozzle to deposit bacterial cells with the desired pattern over a surface to create ELM [81]. Combining bacterial 3D printing technology with an optimised viscoelastic hydrogel, Schaffner et al. [82] demonstrated control over localisation, concentration and composition of bacteria with sub-millimetre accuracy. *B. subtilis* biofilms were also engineered by 3D printing and microencapsulation to produce functional domains useful for several applications in cell biology, enzymology, etc. [83]. A potential drawback of these technologies is the requirement of customised 3D printers. A more affordable technique for 3D patterning of biofilms is MeniFluidics, which exploits meniscus formation within patterns imprinted on a gel surface, allowing patterning colony biofilms in arbitrary shapes with submillimetre resolution [72]. Due to its technical simplicity, MeniFluidics have many potential applications, ranging from patterning bacterial swarms and biofilms to bio-art and tissue engineering.

## Mechanical surface waves and fluid flows control biofilm attachment

Surface undulations and morphological kinetics are also promising tools to control surface colonisation and tailor biofilm morphology. Here, we overview undulatory surface phenomena such as sinusoidal vibrations, pulsations and standing waves that can in fact cause (i) mechanical suppression, (ii) morphological patterning and (iii) hydrodynamic sweeping of surface forming biofilms.

A mechanical inhibition of *P. aeruginosa* biofilm growth has been achieved via the application, over 24 h, of sinusoidal vibrational regimes on polystyrene surfaces at frequencies between 0.2 and 4 kHz and 30 nm in amplitude [84]. The observed inhibition was found to be frequency dependent with minimal biofilm formation at 1 kHz. Because of the homogeneous vibrational displacement and the lack of a significant hydrodynamic effect due to the small nanometric amplitude, the suppression relies mainly on the mechanical interference of vibrations that might falsely activate cellular mechanosensation. In line with these results, sub-micron vibrations (150 nm) at high frequencies (158–168 kHz) for 1hr prevent cell adhesion in *E. coli*, *S. aureus* and *S. epidermidis* [85]*.* However, the response to the vibrations was strain- and material-specific with vibrational effects found to diminish adhesion in *E. coli* for untreated surfaces while adhesion was prevented in *S. aureus* and *S. epidermidis* when surfaces were plasma treated. Antifouling effects on *S. epidermidis* but not on *E. coli* were also observed on piezoelectric elements vibrating at much higher amplitudes (1 mm) and lower frequencies (4 and 40 Hz) after 12 h vibrations [86]. Moreover, upon surface electrical pooling, antifouling properties were preserved at 4 Hz frequency on both positively and negatively charged surfaces while adhesion was instead promoted at 40 Hz. These results confirm the strain specificity of vibrational effects and suggest that these might also depend upon the current electrical state of the vibrated surface.

The promotion and the morphological control of biofilm development was achieved by lowering the frequency and increasing the amplitude of surface vibrations. Standing surface wave patterns (100–1600 Hz and micrometric in amplitudes) on polystyrene surfaces induced by acoustic waves are capable of promoting biofilm formation [87]. Peak biomass, over 48 h stimulation, was observed at 800 Hz and 1600 Hz for *P. aeruginosa* and *S. aureus* respectively [87], with the resulting biofilm morphology showing standing wave-like patterns similar to those originated on the surface. Similar results have been obtained with biofilms in thin liquid layers under surface vibrations attained this time through vertical mechanical displacement [88,89]. For low vibrational frequency (120 Hz), *E. coli* biofilms in thin layers were greatly promoted over 48 h under stable standing wave patterns (2–3 g) with morphologies reflecting those of the wave. Most of the growth was found at waves anti-nodes, suggesting that both QS and mechanosensing may be enhanced at nodes where mechanical stimulation and medium mixing are at their maximum. In contrast with these results, no biofilm growth was observed at higher acceleration (7 g) associated with the turbulent motion of individual oscillations in the thin layer [88]. This finding suggests that biofilm formation and surface colonisation might also be prevented by medium turbulent flow and active mixing, both hydrodynamic effects inducible through surface vibrations. On this regard, the application of sinusoidal wave pulses with variable cycling times, causing millimetric surface displacements, showed outstanding anti-biofilm performances in *E. coli* with maximum decrease in ∼90% surface coverage [90]. The pulse wave movement creates strong hydrodynamic vortexes which, acting as a sweep, interfere with bacteria ability to reach and properly colonising the surface. The fact that undulatory-induced flow can prevent biofilm formation suggests that hydrodynamic properties of the medium can play an important and broader role in biofilms. Indeed, fluid flows can affect not only surface colonisation but also the shape and mechanical properties of the biofilms. This was demonstrated in *Vibrio cholerae* biofilms, where an external shear rate >600 s^−1^ corresponding to a flow speed >10 mm/s led to the formation of more compact biofilms with droplet-like shapes due to shorter cell–cell spacing and elevated production of RbmA attachment protein [91]. This flow also reduced by half the growth rate of biofilm in comparison with low shear rate conditions. External shear flows can also alter the mechanical properties of the biofilm. A study in *S. aureus* identified that an external shear stress of 1–10 mPa makes the biofilm three times harder than the one grown in static conditions, probably as a response to less favourable attachment conditions [92,93].

## Future research directions

In this review, we highlight examples of recent developments on microbial communities in biophysics, bioengineering and synthetic biology. Even though the biological and genetic basis of the multicellular and social behaviours of microbes have been studied for decades, only recently mechanical cell–cell interactions began to be appreciated in the context of microbial communities. Due to the physical nature of the interactions within these communities, we believe that further integration of biophysical studies with biochemical and molecular biological characterisations is the key to the holistic understanding and effective exploitation of collective dynamics.

One important topic of research is the mechanism by which bacterial communities sense and interact with mechanical cues. For example, mechanosensing is coupled to Ca^2+^-dependent membrane depolarisation in *E. coli* [94] and an equivalent coupling has been found between membrane dynamics and neuron membrane potential [95]. Bacterial membrane potential dynamics also mediate electrical signalling in biofilms and during cell differentiation [96–98]. Intriguingly, the coupling between mechanical and electrical dynamics is fundamental to cells due to the physicochemical nature of membranes and proteins [95,99]. While further research is inevitably needed, these findings may suggest that mechanical and electrical control of bacterial communities may offer new biophysical technologies for engineering bacterial collectives as novel functionalized materials.

Another exciting future area of research is bacterial collective information processing. For example, MIPS, which result from physical interactions between active particles, could be an emergent phenomenon underlining a collective decision making in swarming bacteria. Understanding the bacterial collective information processing could lead to developments of dynamic ELM and, moreover, a novel bio-inspired algorithm for swarm robotics, for which developing computational tools is crucial. Equation-based and agent-based modelling are two major approaches, both of which have been successfully applied to model dynamics, behaviours and patterning of biofilms and swarms. There are growing interests in agent-based modelling for rationally engineering and designing complex properties into bacterial communities (see reviews [100–102]). We believe that the integration of computational modelling, soft-matter physics and experimental research focusing on collective dynamics are the key for better understanding and engineering bacterial communities.

## Perspectives

- *Importance of the field*: Bacterial biofilms and swarms exhibit emergent properties arising through local interactions among cells. Emergent properties of bacterial communities provide platforms for developing novel living materials and tools to engineers and synthetic biologists.
- *Summary of the current thinking*: In addition to molecular-biology tools, biophysical understanding and techniques are emerging. Such techniques have the potential of controlling bacterial communities in space and time.
- *Future directions*: In order to rationally design and engineering emergent properties using bacterial communities, further developments of computational tools and biophysical characterisation of cellular dynamics are needed. To this end, we expect to see more interdisciplinary efforts combining microbiology, biophysics, bioelectricity, synthetic biology and computational science approaches.

## Abbreviations

ELM: engineered living materials
EPS: extracellular poly-substances
MIPS: motility-induced phase separation
MSD: mean square displacement
